# Male predominance of pneumonia and hospitalization in pandemic influenza A (H1N1) 2009 infection

**DOI:** 10.1186/1756-0500-4-351

**Published:** 2011-09-10

**Authors:** Won-Il Choi, Byung Hak Rho, Mi-Young Lee

**Affiliations:** 1 Department of Internal Medicine, Dongsan Hospital, Keimyung University School of Medicine, Daegu, Republic of Korea; 2 Department of Diagnostic Radiology, Dongsan Hospital, Keimyung University School of Medicine, Daegu, Republic of Korea; 3 Department of Preventive Medicine, Dongsan Hospital, Keimyung University School of Medicine, Daegu, Republic of Korea

**Keywords:** Influenza, H1N1, Gender, Pneumonia, Admission

## Abstract

**Background:**

Pandemic influenza A (H1N1) disproportionately affects different age groups. The purpose of the current study was to describe the age and gender difference of pandemic influenza A (H1N1) cases that lead to pneumonia, hospitalization or ICU admission.

**Methods:**

Data were collected retrospectively between May 2009 and December 2009. All of the diagnoses of H1N1 were confirmed by real-time reverse-transcription polymerase chain reaction (RT-PCR).

**Results:**

During the study period there were 3402 cases of RT-PCR positive H1N1, among which 1812 were males and 1626 were adults (> 15 years of age). 6% (206/3402) of patients required hospitalization, 3.6% (122/3402) had infiltrates on chest radiographs, and 0.70% (24/3402) were admitted to intensive care unit (ICU). The overall fatality rate was 0.1% (4/3402). The rate of hospitalization was sharply increased in patients ≥ 50 years of age especially in male. Out of 122 pneumonia patients, 68.8% (84 patients) were male. Among the patients admitted to the ICU, 70.8% (17 patients) were male. Approximately 1 of 10 H1N1-infected patients admitted to the ICU were ≥ 70 years of age.

**Conclusions:**

Among the confirmed cases of H1N1, the ICU admission rate was < 1% and the case fatality rate was 0.1%. Male had a significantly higher rate of pneumonia and hospital admission. These findings should be taken into consideration when developing vaccination and treatment strategies.

## Background

The rates of illness from H1N1 virus infection may have variation in relation to geographic variation. During one outbreak in New Zealand, the attack rate of illness was estimated to be 7.5%, and the attack rate of the overall infection was estimated at 11% [1]. The overall case fatality rate has been reported to be < 0.5% [2,3]. The case fatality rate for symptomatic illness has been estimated to be 0.048% in the United States [4] and 0.026% in the United Kingdom [5].

The rates of hospitalization and death have varied widely according to country [6]. In one study, the hospitalization rates have been highest for children < 5 years of age, especially those < 1 year of age, and lowest for persons ≥ 65 years of age [7]. In the United States, among patients who were hospitalized with pandemic influenza, 32%-45% were < 18 years of age [7,8]. Approximately 9%-31% of hospitalized patients have been admitted to an intensive care unit (ICU), where 14%-46% of patients die [7-11]. The overall case fatality rate among hospitalized patients appears to be highest among those ≥ 50 years of age and lowest among children [5,7,11,12].

Studying the case fatality rate, hospitalization cases, pneumonia cases, and ICU cases in relation to age and gender are important for estimating the disease burden and understanding the background of H1N1 virus infections, which in turn may affect treatment and prevention strategies. There is paucity of description of gender difference in hospitalization, pneumonia and ICU admission in pandemic influenza A (H1N1) 2009.

In the present study, we aimed to describe the age and gender difference of pandemic influenza A (H1N1) cases that lead to pneumonia, hospitalization or ICU admission.

## Methods

### Keimyung University Dongsan Hospital (KUDH)

KUDH is a 931 bed tertiary care hospital that also serves as an urban general hospital in Daegu City, South Korea. KUDH serves as a referring hospital an area of about 500,000 inhabitants.

### Collection of specimen

Sterile swabs with cotton tips and wooden shafts were inserted into the oral cavity of the patients. The throat swab was rubbed on the peri-tonsillar surface or the pharyngeal mucosa. The swab was removed and stored in a bottle containing 1 ml of virus transport media.

### Real time RT-PCR assay

RNA was extracted using the Abbott sample preparation system. Viral RNA was reverse-transcribed into complementary DNA (cDNA) using a swine-lineage influenza A (H1N1) generic primer. The subsequent PCR amplification and real-time detection of cDNA was performed on an Abbott m2000rt amplification machine. Each RNA extract sample was tested by the following separate primer/probe sets: InfA; Universal swine (swFluA); Swine H1 (swH1); and RNaseP (RP). The RNaseP primer and probe set targets the human RNaseP gene and thus serves as an internal positive control for human nucleic acid. The test for H1N1 virus was regarded positive if the specimen was positive for the swFluA or swH1 probe, with a concomitant positive reaction to the InfA and RNaseP (RP) probes [13].

### Definition

We defined the symptomatic of influenza as a patient with fever and/or respiratory symptoms and/or generalized symptoms of infection, such as myalgia, headache and chills. We defined fever as a temperature > 38°C. We used Tympanic Electronic Thermometers. The definition of H1N1-associated pneumonia was as follows: 1) symptoms consistent with an influenza-like illness; 2) positive test for real time RT-PCR or RT-PCR; and 3) presence of pneumonic infiltration on simple chest radiography at the time of initial evaluation. There were no strict criteria for general ward or intensive care unit (ICU) admission, but in general patients presenting with several positive SIRS criteria such as heart rate > 100, respiratory rate > 25, and fever > 38°C were admitted to the general ward. Patients had severe hypoxemia or hemodynamic instability were referred to the ICU.

### Data collection

Our study was approved by the Institutional Review Board at Dongsan Hospital of the Keimyung University School of Medicine. Data were extracted from computer-based records of symptomatic patients who were cared for at our hospital due to viral illnesses between 1 May and 31 December 2009. During the study period, our hospital opened specialized H1N1 clinics which were able to assess every patient who had symptoms without the need for referral to other health care facilities. We reviewed all of the medical records of patients with a discharge diagnosis of H1N1 virus infection.

### Statistical Analysis

The rate of H1N1 admission or pneumonia was calculated as the number of admissions or pneumonias per 100 symptomatic H1N1 virus-confirmed patients. Ninety-five percent confidence intervals (CIs) were calculated using the normal approximation to the binomial distribution. A χ^2 ^test was used to compare frequencies. *P *values < 0.05 were considered statistically significant.

## Results

### H1N1 Cases

**Figure 1:**
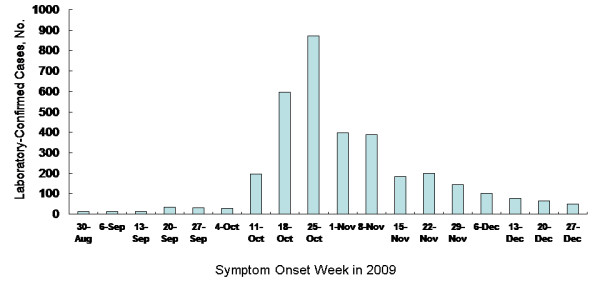
**Symptomatic Patients with Pandemic Influenza A(H1N1) (n = 3,402) Between May 1, 2009 and December 31, 2009**.

Between 1 May and 31 December 2009, 3402 symptomatic pandemic influenza A (H1N1) cases were confirmed out of 11,114 real time RT-PCR tested cases. Among the cases, 1812 (53%) were male and 1590 (47%) were female (Table 1). The median age of all cases was 14 years (range, 1-84 years). After November 1, H1N1 confirmed cases were declined (Figure 1).

**Table 1:** Number of Symptomatic Pandemic 2009 Influenza A(H1N1), Hospitalized, Pneumonia, and ICU Cases by Age and Gender Groups 1 May Through 31 December 2009

	H1N1 positive		Hospitalized		Pneumonia		Intensive Care Unit	
**Age**	**Male**	**Female**	**Male**	**Female**	**Male**	**Female**	**Male**	**Female**

0-4	192	180	27	10	14	6	2	2
5-9	432	311	43	16	33	9	3	0
10-14	373	288	12	6	10	2		
15-19	442	267	9	3	6	3	1	1
20-29	175	219	3	10	1	3		
30-39	85	145	2	5	1	2	0	1
40-49	53	69	7	5	4	4	2	1
50-59	37	58	8	9	7	4	3	0
60-69	15	30	9	9	5	2	3	1
70-79	7	15	5	6	2	2	2	1
80-	1	8	1	1	1	1	1	0
	1812	1590	126	80	84	38	17	7

### Hospitalization

**Figure 2:**
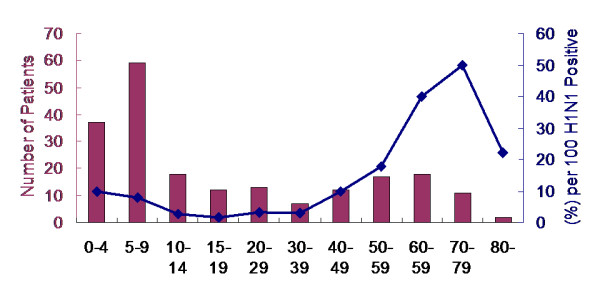
**Number (n = 206) and rate (6.05%) of hospitalizations due to pandemic influenza A (H1N1) infection by age groups among confirmed symptomatic patients**.

**Figure 3:**
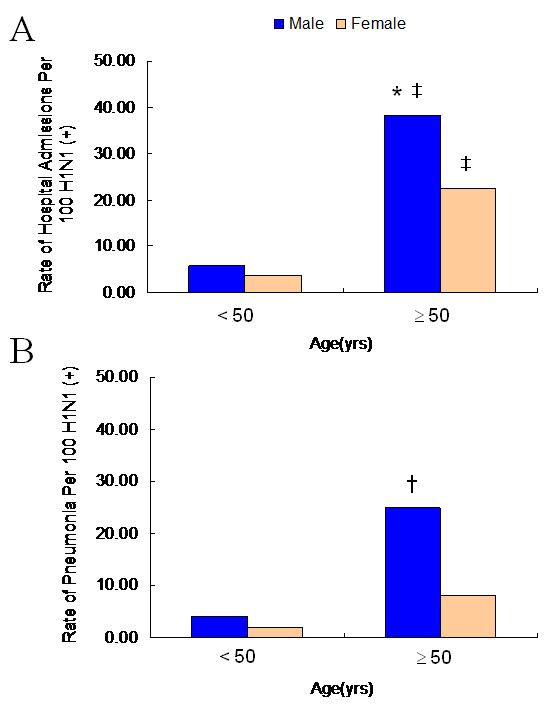
**Rate of hospital admission (A) and pneumonia (B) per 100 pandemic influenza A (H1N1)-confirmed cases**. Results for both male and female are shown separately for those aged < 50 years old and those aged ≥ 50 years old. Comparisons were made of the rate of hospital admission (*P < 0.001) and pneumonia (^†^*P *= 0.0019) between male and female aged ≥ 50. There was a significant difference of the rate of admission between aged < 50 years old and aged ≥ 50 years old in both sexes (^‡^P < 0.001).

The rate of admission was 206 of 3,402 patients (6.05%; 95% CI, 5.3-6.9). The median age of the admitted patients was 14 years (range, 1 - 84 years) and 126 of the patients (61%) were male (P < 0.001) (Table 2). Although the number of hospitalizations (n = 59) was highest in the patients 5-9 years of age, the admission rate was 8% among symptomatic H1N1 patients of the same age. The rate of hospitalization was sharply increased in the patients > 50 years of age (Figure 2) especially in male (Figure 3). Eleven of 22 symptomatic patients (50%) with H1N1 who were admitted to the hospital were ≥ 70 years of age. However, in infants < 1 year and < 1 month of age, the hospitalization rate per 100 symptomatic H1N1 cases was 24% (n = 11) and 82% (n = 9), respectively.

**Table 2:** Comparison of admission and pneumonia between male and female in Pandemic 2009 Influenza A (H1N1) confirmed cases

Number of H1N1 confirmed cases	Male (n = 1812)	Female (n = 1590)	*P *value
Admission	126	80	< 0.001

Pneumonia	84	38	0.006

ICU admission	17	7	0.82

### H1N1-associated Pneumonia

**Figure 4:**
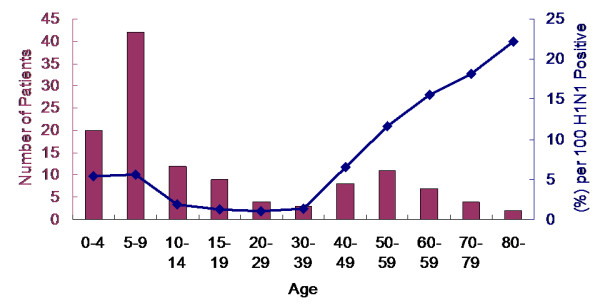
**Number (n = 122) and rate (3.58%) of pneumonia in pandemic influenza A (H1N1)-confirmed patients by age group**.

122 patients (3.6%; 95% CI, 3.0 - 4.2) were diagnosed with H1N1-associated pneumonia among 3402 symptomatic H1N1 patients. The median age of the patients with pneumonia was 9.5 years (range, 1 - 84 years) and 84 of the patients (68.8%) were male (*P *< 0.01) (Table 1, 2). 25 patients (33.7%) reported having asthma among 74 admitted patients < 15 years of age. Ten adult patients (20.8%) who were admitted had asthma or COPD. Although the number of hospitalizations (n = 42) was highest in the patients 5-9 years of age, the rate of pneumonia was sharply increased in symptomatic patients with H1N1 who were > 50 years of age (Figure 4) especially in male (Figure 3).

### ICU admission

**Figure 5:**
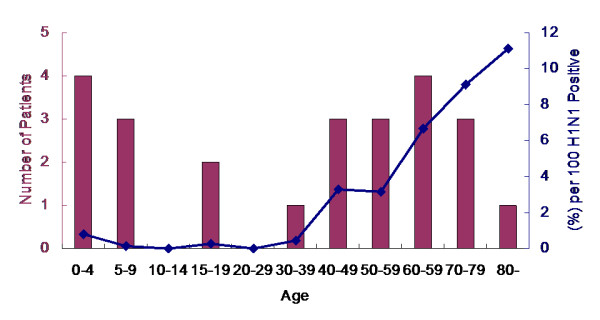
**Number (n = 24) and rate (0.70%) of ICU admissions in pandemic influenza A (H1N1)-confirmed patients by age group**.

Twenty-four patients (0.7%; 95% CI, 0.47 - 0.10) with symptomatic H1N1 infections were admitted to the ICU, and 5 required mechanical ventilation. The median age of the patients admitted to the ICU was 47 years (range, 1 - 84 years) and 17 (70.8%) were male (*P *< 0.05). Ten percent of symptomatic patients with H1N1 admitted to the ICU were ≥ 70 years of age. There were no patients 10-14 or ≥ 20 years of age admitted to the ICU (Figure 5). Of the 206 patients who were hospitalized, 201 (97.5%) received antiviral treatment, and 180 (87%) received treatment within 48 hours of the onset of symptoms. We found no significant differences in the rate of ICU admission between male and female.

### Case Fatality

Four of 3402 symptomatic patients with H1N1 (0.11%) died. All of the patient deaths were ≥ 40 years of age. The mean time from hospital admission to initiation of antiviral treatment was 1.2 days (range, 0-24 days). We found no significant differences in the rate of deaths between male and female.

## Discussion

In the present study, the hospitalization rate, rate of pneumonia, ICU admission rate, and case fatality rate among patients with confirmed symptomatic pandemic influenza A (H1N1) were 6%, 3.6%, 0.7%, and 0.1%, respectively.

Rate of hospital/ICU admission and pneumonia in male were about 2 times higher than female especially in the more than 50 years-old pandemic influenza A(H1N1) 2009 confirmed cases. There was also higher rate of ICU admission in male, while statistics did not reach to meaningful. Different behavior, hormone responses, and susceptibility to infectious disease may lead to distinct profiles of pandemic influenza A(H1N1) 2009-associated morbidity for male and female.

The severity of infection may be measured in a variety of ways; the case-fatality rate, which is the probability that an infection causes death, is the simplest method by which to measure the severity of infection. The case-fatality rate may be influenced by the denominator (the non-fatal patients). Asymptomatic H1N1 virus infections or patients with mild disease were often thought to have a simple cold, and therefore overlooked (undiagnosed). Therefore, diagnostic strategies of the H1N1 virus may have a big influence on the overall fatality rate. The severity of infection may be measured based on the hospitalization cases and ICU cases (the probabilities that an infection leads to hospitalization or ICU admission). The age-specific severity patterns, as estimated herein, are different from the age-specific severity patterns which would be obtained by simply comparing the confirmed cases, hospitalizations, and deaths in the US as a whole for a similar period of time [7,14].

The estimation of the case fatality rate using the laboratory-confirmed cases in the denominator resulted in a rate of 0.82%. This finding is consistent with other studies that have used laboratory-confirmed cases as a denominator [15,16]. The early epidemiologic features of mortality from the pandemic H1N1 infection were different in relation to the region, and the majority of H1N1 virus infection-related deaths occurred in patients 20-49 years of age [15]. The calculated case-fatality rate in Asian countries is lower than on the other continents [15]. The case-fatality rate may have been influenced by the denominator (the non-fatal patients). The mortality rate for symptomatic illness was estimated to be 0.048% in the United States [4] and 0.026% in the United Kingdom [5].

In the present study, the denominator was confirmed symptomatic patients with H1N1; the overall mortality was 0.1%, which was comparable to previous studies [4]. However, the mortality of elderly patients infected with pandemic H1N1 is higher in Asian countries [15]. The elderly appear to be less infected from pandemic influenza A (H1N1), which may be due to a lack of exposure. However, when infected, the elderly are more likely to have a fatal outcome than younger patients [17].

The first pandemic influenza A (H1N1) virus-infected patient in the US was confirmed on 15 April 2009 [18]. Although the first H1N1 patient was confirmed in May 2009, a surge in the number of infected patients was observed 5 months later (October 2009) in KUDH (Figure 1). National H1N1 influenza vaccination program in Korea has started for medical personnel on October 27, and extended in general population on November 1. At the same time with vaccination program has started, H1N1 confirmed cases were declined.

We can confirm all of the H1N1 cases by RT-PCR without delay, even on holidays, and suspected H1N1 cases are treated with oseltamivir before the RT-PCR results are available. These efforts could change the epidemiologic features. The mean time from hospital admission to initiation of antiviral treatment was 1.5 days in the present study, which was more rapid than a previous study [7].

## Conclusion

Among the H1N1-confirmed cases, ICU admissions occurred in < 1% of patients and the case-fatality rate was 0.1%. The rate of pneumonia and ICU admissions in males ≥ 50 years of age have sharply risen. Males had a significantly higher rate of pneumonia and hospital admission especially ≥ 50 years of age. In the present study, the male predominance in relation to rate of admission and pneumonia should be taken into consideration when developing vaccination strategies and empirical treatments are used.

## Competing interests

The authors declare that they have no competing interests.

## Authors' contributions

WIC was responsible for the study design, for data analysis, and for drafted this manuscript; BHR was responsible for the data analysis and interpretation; MYL was responsible for the study design and for data analysis. All authors contributed to the drafting and revisions of the manuscript.

## References

[B1] Baker MG Wilson N Huang QS Paine S Lopez L Bandaranayake D Tobias M Mason K Mackereth GF Jacobs M Pandemic influenza A(H1N1)v in New Zealand: the experience from April to August 2009 Euro Surveill 2009 14 34 10.2807/ese.14.34.19319-en 19712648

[B2] Mathematical modelling of the pandemic H1N1 2009 Wkly Epidemiol Rec 2009 84 34 341 348 19702014

[B3] Wilson N Baker MG The emerging influenza pandemic: estimating the case fatality ratio Euro Surveill 2009 14 26 19573509

[B4] Presanis AM De Angelis D Hagy A Reed C Riley S Cooper BS Finelli L Biedrzycki P Lipsitch M The severity of pandemic H1N1 influenza in the United States, from April to July 2009: a Bayesian analysis PLoS Med 2009 6 12 e1000207 10.1371/journal.pmed.1000207 19997612 PMC2784967

[B5] Donaldson LJ Rutter PD Ellis BM Greaves FE Mytton OT Pebody RG Yardley IE Mortality from pandemic A/H1N1 2009 influenza in England: public health surveillance study BMJ 2009 339 b5213 10.1136/bmj.b5213 PMC2791802 20007665

[B6] Transmission dynamics and impact of pandemic influenza A (H1N1) 2009 virus Wkly Epidemiol Rec 2009 84 46 481 484 19928298

[B7] Louie JK Acosta M Winter K Jean C Gavali S Schechter R Vugia D Harriman K Matyas B Glaser CA Factors associated with death or hospitalization due to pandemic 2009 influenza A(H1N1) infection in California JAMA 2009 302 17 1896 1902 10.1001/jama.2009.1583 19887665

[B8] Jain S Kamimoto L Bramley AM Schmitz AM Benoit SR Louie J Sugerman DE Druckenmiller JK Ritger KA Chugh R Hospitalized patients with 2009 H1N1 influenza in the United States, April-June 2009 N Engl J Med 2009 361 20 1935 1944 10.1056/NEJMoa0906695 19815859

[B9] Dominguez-Cherit G Lapinsky SE Macias AE Pinto R Espinosa-Perez L de la Torre A Poblano-Morales M Baltazar-Torres JA Bautista E Martinez A Critically Ill patients with 2009 influenza A(H1N1) in Mexico JAMA 2009 302 17 1880 1887 10.1001/jama.2009.1536 19822626

[B10] Webb SA Pettila V Seppelt I Bellomo R Bailey M Cooper DJ Cretikos M Davies AR Finfer S Harrigan PW Critical care services and 2009 H1N1 influenza in Australia and New Zealand N Engl J Med 2009 361 20 1925 1934 19815860 10.1056/NEJMoa0908481

[B11] Kumar A Zarychanski R Pinto R Cook DJ Marshall J Lacroix J Stelfox T Bagshaw S Choong K Lamontagne F Critically ill patients with 2009 influenza A(H1N1) infection in Canada JAMA 2009 302 17 1872 1879 10.1001/jama.2009.1496 19822627

[B12] Echevarria-Zuno S Mejia-Arangure JM Mar-Obeso AJ Grajales-Muniz C Robles-Perez E Gonzalez-Leon M Ortega-Alvarez MC Gonzalez-Bonilla C Rascon-Pacheco RA Borja-Aburto VH Infection and death from influenza A H1N1 virus in Mexico: a retrospective analysis Lancet 2009 374 9707 2072 2079 10.1016/S0140-6736(09)61638-X 19913290

[B13] Whiley DM Bialasiewicz S Bletchly C Faux CE Harrower B Gould AR Lambert SB Nimmo GR Nissen MD Sloots TP Detection of novel influenza A(H1N1) virus by real-time RT-PCR J Clin Virol 2009 45 3 203 204 10.1016/j.jcv.2009.05.032 19515611

[B14] Reed C Angulo FJ Swerdlow DL Lipsitch M Meltzer MI Jernigan D Finelli L Estimates of the prevalence of pandemic (H1N1) 2009, United States, April-July 2009 Emerg Infect Dis 2009 15 12 2004 2007 19961687 10.3201/eid1512.091413 PMC3375879

[B15] Vaillant L La Ruche G Tarantola A Barboza P Epidemiology of fatal cases associated with pandemic H1N1 influenza 2009 Euro Surveill 2009 14 33 10.2807/ese.14.33.19309-en 19712643

[B16] Fajardo-Dolci GE Hernandez-Torres F Santacruz-Varela J Rodriguez-Suarez J Lamy P Arboleya-Casanova H Gutierrez-Vega R Manuell-Lee G Cordova-Villalobos JA [Epidemiological profile of mortality due to human influenza A (H1N1) in Mexico] Salud Publica Mex 2009 51 5 361 371 19936549 10.1590/s0036-36342009000500003

[B17] Athanasiou M Lytras T Spala G Triantafyllou E Gkolfinopoulou K Theocharopoulos G Patrinos S Danis K Detsis M Tsiodras S Fatal cases associated with pandemic influenza A (H1N1) reported in Greece PLoS Curr 2010 2 RRN1194 10.1371/currents.RRN1194 PMC2976846 21085493

[B18] Dawood FS Jain S Finelli L Shaw MW Lindstrom S Garten RJ Gubareva LV Xu X Bridges CB Uyeki TM Emergence of a novel swine-origin influenza A (H1N1) virus in humans N Engl J Med 2009 360 25 2605 2615 19423869 10.1056/NEJMoa0903810

